# Proposed Canadian Consensus Guidelines on Osteoarthritis Treatment Based on OA-COAST Stages 1–4

**DOI:** 10.3389/fvets.2022.830098

**Published:** 2022-04-26

**Authors:** Conny Mosley, Tara Edwards, Laura Romano, Geoffrey Truchetti, Laurie Dunbar, Teresa Schiller, Tom Gibson, Charles Bruce, Eric Troncy

**Affiliations:** ^1^Elanco Animal Health, Mississauga, ON, Canada; ^2^VCA Canada, 404 Veterinary Emergency and Referral Hospital, Newmarket, ON, Canada; ^3^VCA Canada, Central Victoria Veterinary Hospital, Victoria, BC, Canada; ^4^VCA Canada, Centra Victoria Veterinary Hospital, Victoria, BC, Canada; ^5^Groupe Veteri Medic Inc., Brossard, QC, Canada; ^6^Montreal Animal Hospital, Montreal, QC, Canada; ^7^Faculty of Veterinary Medicine, University of Calgary, Calgary, AB, Canada; ^8^Grand River Veterinary Surgical Services; Adjunct Faculty OVC, Mississauga, ON, Canada; ^9^Pulse Veterinary Specialists and Emergency, Sherwood Park, AB, Canada; ^10^Faculté de médecine vétérinaire, Université de Montréal, Groupe de recherche en pharmacologie animale du Québec (GREPAQ), Montreal, QC, Canada

**Keywords:** osteoarthritis, physical rehabilitation, weight management, non-steroidal anti-inflammatories, nutra- ceuticals, canine, treatment guidelines

## Abstract

The Canadian consensus guidelines on OA treatment were created from a diverse group of experts, with a strong clinical and/or academic background in treating OA in dogs. The document is a summary of the treatment recommendations made by the group, with treatments being divided into either a core or secondary recommendation. Each treatment or modality is then summarized in the context of available research based support and clinical experience, as the treatment of OA continues to be a multimodal and commonly a multidisciplinary as well as individualized approach. The guidelines aim to help clinicians by providing clear and clinically relevant information about treatment options based on COAST defined OA stages 1–4.

## Introduction

Osteoarthritis (OA) is a challenging disease for veterinarians, patients, and pet owners. The chronicity and disease complexity require extensive education of the pet owner and a willingness to begin a treatment plan for their pet requiring multiple re-assessments over a pet's life dependent on disease progression. The situation is further challenged for veterinarians, as there are a multitude of potential OA treatments, but there is no clear differentiation or priority based on OA stage. It is these understood challenges that led to the specific aim behind the guideline development, to provide prioritized treatment guidance based on clinical experience, with consideration of the available scientific evidence, enabling the Canadian veterinary practitioner to treat and discuss OA based on the different OA stages.

The guidelines are the result of a consensus among a group of Canadian experts in the field of OA including board certified surgeons, anesthesiologists, sports medicine and rehabilitation practitioners, pharmacologist, and general practitioner. The panel members were asked by the lead author to participate in this project based on their clinical expertise, academic knowledge, and active participation in OA education in Canada. A focus for the selection of Canadian members was placed on diversity of fields of interest within OA treatment to represent the clinical, academic and collaborate approach. In the spring of 2021, the panel members virtually met with the goal of creating Canadian specific, OA treatment guidelines based on OA stage. To help frame the initial conversations of the panel, 5 different sample cases were provided ahead of the meeting, with each case representing a typical clinical presentation for the different COAST stage of OA. Each panel member reviewed the cases independently and submitted their approach prior to the meeting. During the meeting, the cases were used to focus the conversation on where treatment approaches were similar or different among the members, discuss specific aspects of the treatment and evaluate the treatment based on scientific support and clinical experience. In addition, topics or challenges that are encountered when treating patients with OA were discussed, i.e., how the panelists approach lowest effective dose, challenges in pain assessment.

After the case discussions finished, the panel moved on to discuss more generally, how to adjust the treatment approach based on the different COAST stage. Each treatment was then evaluated and voted on. In order for a treatment to be classified as “core” it required 9/9 agreement. Therefore, core treatment recommendations were unanimously agreed on to be included in any case with OA with specific nuances adjusted to the different stages and individual patient. If a treatment did not receive unanimous support, it was classified as secondary, and then further discussions occurred as to at what stage, and when the treatment should be considered. The secondary treatments received varying levels of support due to the often lack of available research for a particular treatment, and instead those in favor of the treatment, provided their clinical knowledge and experience. A consensus was reached for when to start the secondary treatment options based on the COAST stage, however, there was no priority assigned (which order to start treatment A, then B, etc.) and instead the treatments were simply grouped. Overall, the authors focused on available or soon to be available treatment options in Canada.

In human medicine, chronic pain guidelines are based on evidenced based medicine and therefore backed up by extensive scientific studies, that provide appropriate evidence. In veterinary medicine such work with clear evidence is unfortunately not available in chronic pain management. The limitations are mainly due to inadequate objective pain assessments, and knowledge gaps remain within most treatment options, despite many efforts from well-performed studies.

This review article summarizes the consensual guideline results, that were based on the shared opinions of the Canadian experts using evidence-considered treatment information and their own clinical experience. Compared to the classical evidence based approach adopted in human clinical guidelines, the scope of this review is therefore more narrow in focus, documenting the scientific, and clinical insights of the panel members. Within the description of each treatment option, a focus was placed on explaining the mechanism of actions and pharmacology of each treatment to increase the reader's knowledge and understanding of its benefits or limitations as a potentially effective treatment in canine OA. The literature citing reflected this focus.

## Osteoarthritis

Osteoarthritis is the most common joint disease affecting dogs. Most papers reference that ~1 in 4 dogs are affected (1–3), although it has been suspected that this number may be an underestimation due to this disease being underreported until later stages (3). It has to be mentioned that the actual original studies that continue to be referenced are either older, have a small sample size, or represent a very specific regional selection, among other limitations, and show a variety of OA prevalence results (4–6). Osteoarthritis is a disease of the entire joint organ with loss and dysfunction of the articular cartilage and is usually highly inflammatory in nature. Resulting changes will progressively impact all structures within the joint, including a thickened joint capsule with inflamed synovium and reduced viscosity of synovial fluid, damage to cartilage and subchondral bones, and development of osteophytes. The etiology of OA is complex with local mechanical as well as systemic and metabolic contributing factors (7–9). The chronic and progressive characteristics make it a challenging disease for clinicians to control. In addition, the treatment recommendations in the literature can be inconsistent and vague, and the clinical approach often varies among veterinarians. The individual case response, including both patient and client variability, adds to the complexity when making decisions for a treatment plan. The age of the dog and the different stages of the disease further impact treatment decisions and create more confusion due to inconsistencies in recommendations. When left untreated, OA can progress to a severe debilitating disease with significant functional impairment and pain sensitization. Early detection of OA and early treatments are considered important aspects in slowing down the progression of the disease and enhancing the quality of life (QoL) of the pet.

**Figure 1:**
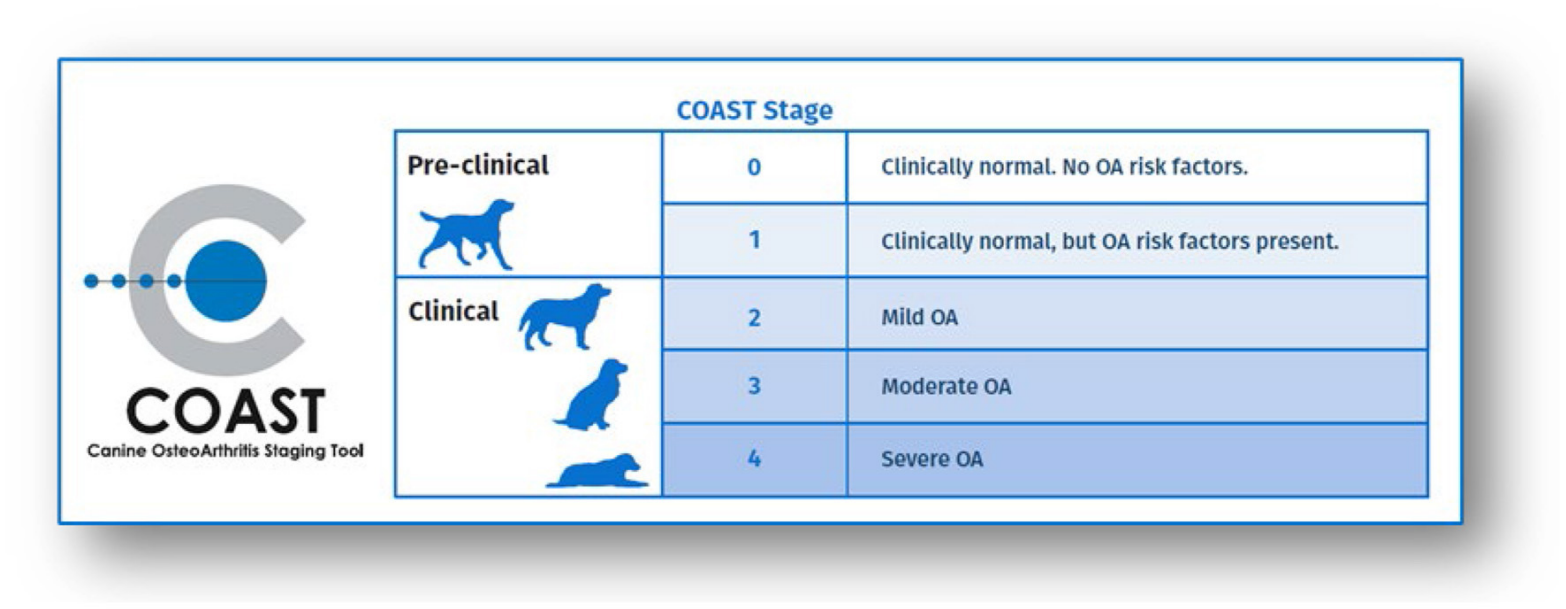
OA stages based on Canine OsteoArthritis Staging Tool (COAST). Image courtesy of Elanco.

Regarding identifying the patient's stage of OA, the Canine OsteoArthritis Staging Tool (COAST) is a helpful diagnostic tool to assist veterinarians -with input from pet owners-, in recognizing and treating canine OA from its earliest stages (10). The tool provides clear guidance on how to decide on a dog's current OA stage based on owner input, orthopedic exam, and radiographic findings. The COAST stages include 0 (clinically normal, no risk factors), 1 (clinically normal, but OA risk factors present), 2 (mild OA), 3 (moderate OA), 4 (severe OA) (Figure 1). The descriptions of each stage are included in the category discussions below.

With OA, it is important to identify risk factors early in the disease and intervene before significant clinical signs occur, with the goal of preventing and/or slowing the progression (11). For consistency and to ensure clear definitions of each stage, these treatment guidelines are based on the COAST definitions of OA stages 1–4. We have not included Stage 0 as a stage that requires treatments but recognize that due to the high prevalence of OA in dogs, it is important for veterinarians to provide OA risk factor and prevention education at an early age even in this stage. The general education and Stage 1 discussion points also apply to Stage 0.

## Treatment Guidelines

### General Treatments Regardless of Stages

For all stages of OA, **client education** is fundamental. The veterinarian's role to educate owners on the disease (including pathology, risk factors, progression, stages, and identification and recognition of pain behaviors) is crucial. Education also includes relevant components like nutrition, specific diets, weight management, regular assessments, and therapeutic options (pharmaceutical, nutraceutical, physical medicine modalities, importance of exercise, lifestyle changes, and home improvements).

Osteoarthritis is a painful disease that results in limitations to the dog's ability and can progress to being severely debilitating. Education and empowering owners to recognize and identify the early signs of pain will help with early detection and treatment of the disease. Signs of pain in the later stages can help evaluate treatment response as well as its impact on the QoL of the pet. Signs in the early stages (Stages 1 and 2) can be subtle like asymmetric posture when standing or sitting, slight difficulties in rising or laying down, reluctance to jump into car, reluctance to play, young dogs not able to keep up with others, and difficulties with stairs. Pain in the later stages of OA is described below in their specific stage (Stage 3 and 4).

For general treatment recommendations, one common denominator in all OA stages is **weight management** (reaching and maintaining an ideal body condition score) (12–15). Obesity has been considered a high-risk factor for the development and progression of OA. Historically, this was ascribed to the excessive biomechanical joint loading on the articular cartilage, because of increased body weight, causing micro injuries and subsequent wear and faster breakdown. However, the association between obesity and OA in non-weight-bearing joints suggests a more complex etiology for obesity-induced OA. A more important part of the pathogenesis of OA could be the systemic and metabolic effects of obesity (inflamed adipose, dyslipidemia) (16, 17). Fat produces systemic inflammatory factors (cytokines and adipokines), which are specific adipose tissue-produced factors with significant inflammatory properties (18), which we presume from other species' extrapolation is also the case in dogs. The influence of adipose tissue attributing to low-grade systemic inflammation has been recognized and a weight loss program has shown both in humans and dogs to have general health benefits and potentially decrease and slow down the progression of OA in humans (19) and dogs (15), and is therefore considered by many an actual treatment option (20). Thus, an increase in body weight has been demonstrated to have negative effects on the osteoarthritic joint load (21) and maintaining optimal body condition should be one of the most important goals for any patient at any OA stage in the opinion of the panel. A specific effort should be made to educate and support owners in a weight reduction plan for their pet. This includes nutrition counseling for the right diet (weight or joint health focused or both), including both caloric and omega 3 fatty acid (FA) dose recommendations. This can also be used for a weight maintenance plan throughout the patient's life.

Dogs with OA require **regular exercise**. This is an important aspect of OA management for dogs. Exercise may be modified depending on the disease stage, but it is crucial that dogs with OA maintain a regular exercise plan that limits high impact and torsion to minimize joint trauma to help keep the joints mobile, cartilage healthy, and maintain muscle strength to support the joint (22–25). Historically “prolonged rest” was prescribed in cases with OA pain. This approach has the disadvantage that when a joint lacks movement, it will stiffen further (fibrosis) and decline cartilage health (26). A lack of exercise will contribute to muscle atrophy, thereby further reducing joint stability and contributing to pain (27, 28). The practice of severe activity restriction or rest is generally not recommended, instead regular, low impact exercise is an important part of pain management in OA. Regular physical activity is crucial to slow down the progression of sarcopenia and maintain physical fitness in dogs with OA (29), including the geriatric population. The specific type and frequency of exercise is dependent on the different stage of disease as well as the joints affected. Examples of low impact exercises could be frequent daily leash walks and a program with specific or targeted therapeutic exercises.

### Specific Recommendations for the Different Stages

Recommendations for specific stages of the disease will be presented below and are useful starting points for most animals at each stage.

Serial monitoring of these patients is necessary, and treatment should be adapted according to the patient's response. Please note that multiple joints can be affected, and each joint may be in a different OA stage. There was agreement among the expert panel that targeting the joint with the worst OA stage will ensure an appropriate treatment plan for the patient. This was in agreement with the COAST reference of OA staging (10).

Please note, when reviewing the treatment guidelines, the below considerations may require adjustment of the therapeutic approach:

- Multiple joints affected requiring specific targeted therapies for an individual joint.
- Additional co-morbidities or concurrent medications present.
- Adverse events encountered in response to therapy.
- Surgical therapies were beyond the scope of the guidelines, please consider surgical interventions as appropriate for the patient.

Some of the suggested treatments are not licensed for the use in dogs or may have limited scientific evidence specifically for OA in dogs. It is the veterinarian's duty to make a risk:benefit assessment for each patient prior to administering any treatment and provide all relevant information related to the treatment.

### Coast Stage 1

Stage 1 refers to a patient that is currently normal (preclinical) but has risk factors for developing OA (10). Based on the COAST literature, our panel identified that risk factors may include a genetic predisposition, extensive, and longterm participation in injury prone activity, a joint injury or surgery, and excess body weight or age. A typical COAST Stage I dog would be a specific breed with atypical limb conformation either breed related (i.e., Basset Hound, Bulldog, German Shephard etc.) or congenital/traumatic deformities (elbow/hip dysplasia; giant breed dogs) that could cause abnormal joint loading. The treatment is focused on the prevention of the disease.

#### Treatment Goals

Provide adequate education to owners about the high prevalence and risks of canine OA as well as early recognition and clear prevention measures. Maintaining joint health is a priority in this stage. If a joint injury or surgery are contributing factors, the importance of effectively controlling inflammation and pain in the peri-and post surgical/injury time is imperative.

#### Prioritized Treatment

For Stage 1, client education begins with a strategy for growing puppies, including nutrition, weight management and exercise, as stated above. More specific education for this stage include education on the risk factors, as well as guidance for specific training and exercises for injury prevention. Owners of working and sporting dogs may especially need a reminder about the importance of regular musculoskeletal assessments for early recognition of OA.

#### Diet and Omega 3 Fatty Acids

In dogs with a higher risk factor for OA, a diet with focus on joint health is ideal to ensure that the dietary ingredients included support the musculoskeletal system. In particular, omega 3 FAs have shown to be effective in reducing the signs and progression of OA (30–38), although it is important to provide adequate dosing (39). Most joint health focused diets have omega 3 FAs at varying dosing ranging from 0.59 to 10.11 g/1,000 kcal, with many brands being under 3.5 g/1,000 kcal (40). It is imperative to identify the actual eicosapentaenoic acid (EPA) and docosahexaenoic acid (DHA) concentrations in the food, as not uncommonly alpha lipoic acid (ALA) is used in foods for its omega 3 FA content. As described below ALA is not an adequate substitute and food should quantify actual levels of DHA and EPA. In most cases, additional DHA/EPA supplementation is required to reach the scientifically recommended minimum dose of 100 mg/kg daily DHA/EPA (32). The type of omega 3 FA supplementation for adequate conversion to DHA/EPA should be based on current scientific evidence. The precursor of DHA/EPA in plants is ALA. The conversion rate from plant-based ALA to EPA is significantly less than from fish/marine based oil and a full conversion from ALA to DHA does not occur, only to its precursor docosapentaenoic acid (DPA) (41, 42). Higher conversion rates with significantly more reduction of inflammatory markers were found with fish/marine based oils. In some patients, the joint health focused diet may need to be assessed for calories to reflect weight management goals and activity levels. Weight optimization is recommended throughout the patient's life as mentioned above.

#### Rehabilitation

Depending on the risk factors for the dog, a rehabilitation veterinarian can be sought out to discuss disease prevention, strategies to slow down progression of disease, and recommend therapeutic exercises to promote strengthening of muscles supporting joints. For an athletic or service dog, adequate training tips for injury prevention may be beneficial (*i.e*., focusing on strength, endurance, proprioception, limiting repetitive, and concussive activity). For a dog with a genetic or breed specific predisposition, specific exercises could be useful to implement into daily activities (43).

These consults by a veterinarian or rehabilitation practitioner may also include lifestyle and household modifications, for example, daily exercises (including walks, swims) or household modifications (early teaching of a young Dachshund not to jump on and off a sofa, adding in a step stool, improving flooring traction, etc.).

A summary of stage 1 treatment recommendations is provided in Table 1.

**Table 1:** Summarized core treatment recommendations for COAST Stage 1.

**STAGE 1**	
**Core treatment recommendations**	
Client education	Risk factors identification, disease prevention, assessment plan
Weight optimization and nutrition	Adequate DHA/EPA supplementation, joint focused diets
Regular exercise	Well-balanced training and injury prevention
Physical rehabilitation	Injury prevention strategies, risk factor identification, muscle strength support

### Coast Stage 2 (Mild OA)

Stage 2 refers to the early clinical stage of osteoarthritis that results in mild clinical signs. Those signs can be inconsistent and subtle, can occur with some activities or after activities, may affect the gait and show subtle changes/shifting in body weight distribution and limb loading. Range of motion (ROM) of a certain limb/joint may be minimally reduced, but crepitus is unlikely at this stage. Minimal osteophytes and early signs of OA may be visible on diagnostic imaging (10).

#### Treatment Goals

The treatment goals at this stage are supporting the preservation of healthy cartilage and treating flare ups promptly and effectively. Providing owner education on recognizing signs of OA and importance of early as well as longterm treatment can be teadious, but is needed for the desired compliance.

#### Prioritized Treatment

Client education encompasses all points mentioned in the general treatment recommendation section as well as the Stage 1 specific education points.

A joint health focused diet and weight optimization are recommended as mentioned in the general section. DHA/EPA at a minimum of 100 mg/kg daily dose should be included within the diet or additionally supplemented.

Further important Stage 2 discussion topics include the progression of OA from mild to moderate stages, the importance of regular orthopedic assessments and monitoring response to therapy, as well as developing an exercise program suited for the patient. Daily exercise is necessary and should be low to moderate impact, for example walks, swims or specific physical exercises as recommended (43, 44). Exercises with high impact or torsion, like ball throwing, should be avoided. A specific fitness and exercise plan is necessary to be set in place for working dogs that require to return to work. This plan would be based on the type of work, the type of joint/dog concerns, and should focus on further injury prevention.

A consultation with a rehabilitation practitioner (when possible) would be beneficial to identify factors that may contribute to the faster progression of the disease and help with tips on how to decrease risk factors and optimize muscle strength, posture, proprioception, and gait. An initial assessment of gait, weight bearing, transitions, posture, body condition score, muscle condition score, ROM, and pain scoring provides a baseline evaluation of musculoskeletal health. Understanding what areas need improvement allows for a more individualized treatment plan. Targeted therapeutic exercises may focus on core strength and posture, maintaining or gaining ROM, improving overall physical fitness, and strengthening the musculature that is required to provide stability for arthritic joints (28, 45). Specific exercises should be prescribed depending on location of arthritis, concurrent illness, pain level, temperament/trainability of the dog, physical limitations of the owner, physical strength and endurance of the dog, home environment (i.e., condo vs. farm dog) and it is beyond the scope of this paper to address the multitude of situations. In addition, there are many options including manual therapy, physical medicine modalities, and rehabilitation equipment that can be utilized to treat and manage the arthritic patient. A rehabilitation program at this stage may include a combination of both specific home exercises and a formal in-clinic rehabilitation program.

As part of the client education or the rehabilitation consult, lifestyle and household modifications should be included at this stage. These may incorporate modifications in the house (stairs, flooring) or car (adjusted jumping out or in) to prevent high impact injuries and start learning/switching habits for future mobility concerns.

For **pain management**, the use of **non-steroidal anti-inflammatory drugs (NSAIDs)** is warranted for this stage, as a patient is demonstrating clinical signs. Due to the inflammatory nature of OA especially at the early stage (46, 47), NSAIDs play a significant role in decreasing the pathogenesis of peripheral sensitization. Prostaglandins (PG), in particular PGE_2_, are one of the main inflammatory mediators in arthritis and will contribute to the transition from acute to a maladaptive chronic pain state when untreated (48, 49). Therefore, NSAIDs are considered to be the cornerstone of rheumatoid arthritis and OA treatment, providing effective pain relief, especially in this initial clinical stage by inhibiting cyclooxygenase (COX) activity and subsequently blocking the production of prostanoids, including PGE_2_ (50, 51). When the production of this prostaglandin is increased in response to an inflammatory event, PGE_2_ is more readily available to bind to its specific receptor [E-type prostanoid receptor 4 (EP4 receptor)] on the presynaptic side at the site of inflammation, resulting in a pain signal to be sent across the synapse and subsequently travel up the pain pathway. Without interference and continuing phasic and/or static nociceptive input, the constant stimulation from PGE_2_ *via* the EP4 receptor pathway will lead over time to an increase in the sensitization of sensory neurons. The EP4 receptor will be upregulated in a prolonged state of inflammation (52). Upregulation means that the receptors are in a higher state of alert and increase in number. This constant activation results in further pain and increased inflammation. The ability to dampen the receptors (piprant class NSAID) or decrease available inflammatory prostaglandins (COX-2 selective inhibitory NSAIDs) will decrease the pain sensitivity and contribute to limiting sensitization (49), that could lead to chronic maladaptive pain.

The response to NSAIDs can be individual and the right fit regarding efficacy, adverse events, and predicted duration of use should determine the choice of a specific NSAID for the patient. Considering the importance of NSAIDs in the disease of OA, sometimes a patient may need to switch to a more suitable NSAID after the appropriate washout period (53, 54).

For dogs, this panel all agreed on a NSAID trial for a minimum of 4 weeks at product's labeled dose. The minimum of 4 weeks is recommended to allow for an adequate decrease of inflammation (55). Improvements of clinical signs may show earlier than 4 weeks, but the consensus is to treat for the full duration of the NSAID trail to resolve the inflammation on a cellular level. As most adverse events commonly occur in the early phase of initial treatment, a “check-in” call after 1 week to discuss the patient's tolerance and acceptance of the medication is recommended. After the 4-week trial, the patient should be reassessed clinically and, based on therapeutic response, the treatment can be continued or discontinued as appropriate. Not uncommonly it is recommended to continue NSAID therapy long-term to allow for effectively treating the underlying inflammatory nature of OA at this stage. With long-term NSAID use, bloodwork is recommended with a baseline CBC/Chemistry prior to initiating NSAID treatment and then every 3–6 months as needed, unless concerns about the health of the dog arise earlier.

“Flare-ups” (also known as “acute-on-chronic-episodes”) can occur because of activity, injury, weather, and should aim to be minimized (56). In the occurrence of a flare up with clinical signs, if NSAIDs are not currently being used in the patient, a trial should be most strongly considered to keep the inflammation at a minimum as currently the only product proven to be effective to achieve this are NSAIDs. Median duration of flare-ups was reported in humans to last 5 days (56). In dogs, our recommendations for the NSAID treatment during an aggravated, more obvious painful period, would be at a minimum of 3–5 days -or longer- until resolved, with the oversight of a veterinarian. A patient with re-occuring flare-ups should remain on long-term administration of NSAIDs to reduce inflammation, that will lead to sensitization and maladaptive pain. The benefits of a more chronic NSAID dose regimen outweighs the perceived risks (54). Owner education on this aspect is necessary to negate potential reservations and increase compliance.

Similar to the above, patients should always be assessed for improvement and monitored for any potential adverse events.

#### Secondary Treatment Options

Within stage 2, there is a high variability in case presentation. Depending on the presenting clinical signs, secondary treatment options should be tailored to each individual patient on a case-by-case basis. No consensus was reached among the panel members on specific treatment recommendations due to limited evidence and differing clinical approaches resulting from the inconsistency of case presentations. Nevertheless, due to the progression of OA, the development of chronic pain, or acute on chronic flare ups, adjunctive pain medications or treatment modalities may be required. Physical medicine modalities that focus on reducing inflammation and managing pain are suggested to be considered on an individual basis (i.e., photobiomodulation, pulsed-electromagnetic field therapy (PEMF), acupuncture, cryotherapy). Implementing a long-term joint health plan with chondroprotective products is aiming to slow down the progression of the disease, but scientific evidence of efficacy for canine OA is currently still limited. The choice of recommeded chondroprotective product is dependent on the specific case presentation.

A summary of stage 2 treatment recommendations is provided in Table 2.

**Table 2:** Summarized core and secondary treatment recommendations for COAST Stage 2.

**STAGE 2**	
**Core treatment recommendations**	
Client education	Disease and progression, assessment and treatment plan
Weight optimization and nutrition	Adequate DHA/EPA supplementation, joint focused diets
Regular exercise	Well-balanced training and suitable daily exercise
Physical rehabilitation	Injury prevention, risk factor identification, muscle strength support
Pain management	NSAIDs, flare up reduction
**Secondary treatment considerations**	
Chondroprotective joint health support	Additional supplements for joint support

### Coast Stage 3 (Moderate OA)

Stage 3 refers to the clinical stage of OA that results in moderate clinical signs and moderate signs of discomfort. Those signs are more consistent and obvious at all gaits and activities, with consistent clinical abnormalities. There are noticeable changes in body weight distribution and limb loading and obvious reduction in use of affected limb(s). Some difficulties in rising or laying down are present. A decrease in ROM is present and muscle atrophy can be seen. Joint thickening may be noticeable. Obvious osteophytes and signs of OA are likely evident on diagnostic imaging (10). This is the stage that most dogs are presented for orthopedic and/or pain evaluation.

#### Treatment Goals

At Stage 3, the treatment goals are an individualized and effective treatment of these multi-facetted pain states and maintaining a tailored level of mobility for the specific patient based on both patient and client. This includes specific interventions aimed at slowing the OA progression and mobility decline.

#### Prioritized Treatments

**Client education** includes all the topics mentioned in the general treatment recommendations for nutrition, weight management, exercise, and regular reassessments.

Specific focus points for education at Stage 3 would be the progression of disease, impact on quality of life, and appropriate pain management. The importance of regular assessments should be emphasized to allow for tracking musculoskeletal changes and response to treatment. Individualized home exercise programs, lifestyle adjustments, and household modifications will require adjustments over time.

At Stage 3, a **formal rehabilitation program** designed by a rehabilitation practitioner is highly recommended if logistics allow. Rehabilitation can ensure appropriate assessment and treatment of pain on a regular basis, aiming to slow down the disease progression with a focus on mobility. A rehabilitation partnership provides support to owners for their dog's debilitating disease. This support can include QoL assessments and discussions. A rehabilitation team will create an individualized program for the patient that may include targeted therapeutic exercises which focus on core strength and posture, maintaining or gaining range of motion, improving overall physical fitness, and strengthening the musculature that is required to provide stability for arthritic joints (44). A rehabilitation team should use the fundamentals of rehabilitation to create a long-term rehabilitation plan that considers the dog's and owner's desired lifestyle. The plan should be patient-centric and based on canine physiological and scientific principles. The dog's initial presentation and progress is based on individual assessment. It must address the degree of tissue damage and healing, pain experienced in rest and during exercise, strength, and desired functional goals. An understanding of the phases of tissue healing, frequent patient reassessments, and clinical reasoning skills to progress treatments appropriately for the individual patient are the cornerstones of a successful rehabilitation program (57). A rehabilitation program at this stage often includes a combination of a home exercise plan in addition to a formal in-clinic rehabilitation program. Effective pain management is the fundamental basis for a successful rehabilitation program and contributes to patient compliance and owner motivation.

**Lifestyle and household modifications** play an important role at this stage to prevent injury and improve QoL by simplification of obstacles. Examples may include ramps for easier access to stairs/car, baby gates to block off stairs for prevention of falls/injuries, carpet runners or yoga mats over slippery floor to prevent slipping, well-padded dog beds for easier comfort, improved traction with nail covers or grips to prevent slipping and dragging toes, assistive devices such as special harnesses (Help'emUp harness) for improved mobility. Adequate nail trimming is also an underestimated tool to assist with proper biomechanics and appropriate alignment. Improving traction and reducing risk of slipping is further achieved by appropriate trimming of foot fur to allow for pads contacting the floor.

For **pain management**, the use of **NSAIDs** is highly recommended at this stage. NSAIDs are the cornerstone of providing adequate anti-inflammatory therapy for OA (54). The initial protocol is very similar to the stage 2 NSAID description. However, if the response of the 4-week trial is showing favorable results for the pet, the use of NSAIDS will most likely be required on a long-term basis. Most dogs tolerate the long-term use of NSAIDs well, although regular wellness visits, bloodwork, and treatment reassessments are needed.

Long-term use of NSAIDs may produce some questions or concerns from both owners and veterinarians (54, 58). NSAIDs have proven the most effective medication for OA but administration does carry the potential risk for adverse events (gastrointestinal, renal) in particular with patients with pre-existing risk factors. Most common adverse events described in dogs appear to be gastrointestinal related and that is a common cause of concern for veterinarians and pet owners alike (53, 54, 58). In human medicine, it has been recommended to use the lowest effective dose for the shortest time possible (59), but the challenge with this recommendation is the risk for suboptimal pain relief with non-verbal patients in veterinary medicine. One study evaluated the efficacy of ketoprofen at lower than label dose in an acute experimental inflammatory model using a weight bearing assessment tool with results showing analgesic efficacy compared to the control group (60), but overall clinical studies are limited addressing the dose reduction approach. Concerns are that lowering the dose can be quite problematic, considering the limitations of owners (and veterinarians in clinic settings) to adequately assess pain, specifically subtle changes. The need for studies assessing if using concurrent medications that may work synergistically with NSAIDs due to similar pathways are needed to evaluate the potential for dose reductions. A canine study on the non-selective COX inhibitor ketoprofen (61) showed that reducing the recommended NSAID dose by 75%, significantly reduced the measured side effects (glomerular filtration rate, gastro-intestinal lesions) but not platelet aggregation changes, and the reduced dose did provide some OA pain relief, although this was improved in conjunction with tramadol (5 mg/kg/day PO, slow-release formulation). The comparison of pain scores to the group given ketoprofen at the label dose was unfortunately not presented. A similar dose-reducing study compared a reduced dose of meloxicam (62) to the recommended label dose, and it concluded that the adequacy of pain control was lower with the reduced dose. This study gradually reduced the dose over time (15% reduction) every 2 weeks. Only the first 15% reduction was tolerated by the majority of the dogs (87%), while further reduction revealed inadequate pain control in some dogs (62). This led the authors to conclude that a small dose reduction may maintain efficacy but does not seem to be consistent and appears to be based on individual responses. This may be difficult to differentiate clinically and will require the owners' ability to appropriately assess pain. The study found minimal adverse events in the recommended label dose group of the study over a period of 100 days. See Table 3 for OA approved NSAIDs in Canada.

**Table 3:** Non-steroidal antiinflammatory drugs available in Canada with label indication for OA.

**Generic name**	**Brand name**	**Manufacturer**	**Indication**	**Size(s)**	**Dose**
Carprofen	Rimadyl	Zoetis	Relief of pain and inflammation in dogs and relief of signs associated with osteoarthritis.	25, 75, and 100 mg Tablets	4.4 mg/kg PO q 24 h or 2.2 mg/kg PO q 12 h
Deracoxib	Deramaxx	Elanco	Treatment of chronic pain and lameness associated with osteoarthritis.	25, 75, and 100 mg Tablets	1–2 mg/kg PO q 24 h or LED
Firocoxib	Previcox	Boehringer Ingelheim	Control of pain and inflammation associated with osteoarthritis.	57 and 227 mg Tablets	5 mg/kg PO q 24 h
Grapiprant	Galliprant	Elanco	Treatment and control of pain and inflammation associated with osteoarthritis in dogs.	20, 60, and 100 mg Tablets	2 mg/kg PO q 24 h
Meloxicam	Apo-Meloxicam	Apotex	Alleviation of inflammation and pain in both acute and chronic musculoskeletal disorders (dogs).	1.5 mg/mL Suspension	Day 1: 0.2 mg/kg PO q 24 h Maintenance: 0.1 mg/kg PO q 24 h
Meloxicam	Inflacam	Virbac	Alleviation of inflammation and pain in both acute and chronic musculo-skeletal disorders (dogs).	1 and 2.5 mg Tablets	Day 1: 0.2 mg/kg PO q 24 h Maintenance: 0.1 mg/kg PO q 24 h
Meloxicam	Meloxadin	Vetoquinol	Alleviation of inflammation and pain in both acute and chronic musculo-skeletal disorders (dogs).	1.5 mg/mL Suspension	Day 1: 0.2 mg/kg PO q 24 h Maintenance: 0.1 mg/kg PO q 24 h
Meloxicam	M-Eloxyn	Zoetis	Alleviation of inflammation and pain in both acute and chronic musculo-skeletal disorders (dogs).	1.5 mg/mL Suspension	Day 1: 0.2 mg/kg PO q 24 h Maintenance: 0.1 mg/kg PO q 24h
Meloxicam	Metacam	Boehringer Ingelheim	Alleviation of inflammation and pain in both acute and chronic musculo-skeletal disorders (dogs).	1.5 mg/mL Suspension 1 and 2 mg Tablets	Day 1: 0.2 mg/kg PO q 24 h Maintenance: 0.1 mg/kg PO q 24 h
Meloxicam	Rheumocam	Merck	Alleviation of inflammation and pain in both acute and chronic musculo-skeletal disorders (dogs).	1.5 mg/mL Suspension	Day 1: 0.2 mg/kg PO q 24 h Maintenance: 0.1 mg/kg PO q 24 h
Robenacoxib	Onsior	Elanco	Control of pain and inflammation associated with osteoarthritis in dogs.	5, 10, 20, and 40 mg Tablets	1–2 mg/kg PO q 24 h

Although all approved NSAIDs in Canada provide recommendations to utilize the lowest effective dose, assessing the adequate efficacy for appropriate pain control remains a significant challenge. Utilizing client-based questionnaires, e.g., Liverpool Osteoarthritis in Dogs (LOAD), can help raise owner's pain recognition awareness and aid in assessing the response to treatment (63).

#### Secondary Treatment Options

Secondary treatment options are often needed at stage 3 and 4 due to the difficult characteristics of OA pain. Depending on the presenting clinical signs, secondary treatment should be tailored to each individual patient on a case-by-case basis. The pain experience is unique for every individual, as is their response to treatment(s). Factors including a patient's personality, receptor genetics, metabolism, and degree of peripheral and central sensitization, which all serve to emphasize the importance of tailoring treatments to an individual patient.

The multimodal approach can be confusing due to the multitude of options, limited evidence in some instances, and the high variation in individual response in efficacy.

No unanimous consensus was reached among the panel members on secondary treatment recommendations due to limited or variability in evidence, therapy available and differing clinical experiences.

Instead, a summarized review of the most common secondary treatment options are provided.

The order of what, when and how to introduce a new secondary medication or modality to the multimodal approach is dependent on the individual dog, owner, veterinarian, and availability (of modality). In this following section a brief summary of options for Stage 3 specific is provided (with more detailed description of those treatment options listed below the Stage 4 category as all options may be relevant for both Stages 3 and 4).

- Gabapentin or pregabalin are usually added as a second line treatment based on the clinical experience of some panel members, when the core treatments are not sufficient to control the patients clinical signs. The evidence for the use of gabapentin (or pregabalin) for OA is limited to non-existent, although it is considered a good additional medication when a neurogenic/neuropathic component is expected (see detailed description in appendix, including the advantages of pregabalin over gabapentin).
- Photobiomodulation (64) and acupuncture (65) are considered appropriate modalities to support the multimodal therapy approach based on subjective outcome measures and clinical experience of some panelists; see detailed description in appendix.
- Some panelists would consider joint injections with platelet rich plasma (PRP) or hyaluronic acid (HA)/triamcinolone at this time if a particular joint is refractory to treatment (66–68).
- Some panelists would consider cannabinoids at this time with veterinary oversight for close monitoring and appropriate selection of a suitable quality product (69, 70).

A summary of stage 3 treatment recommendations is provided in Table 4.

**Table 4:** Summarized core and secondary treatment recommendations for COAST Stage 3.

**STAGE 3**	
**Core treatment recommendations**	
Client education	Disease progression, regular assessment and adequate treatment plan, QoL, pain management
Weight optimization and nutrition	Adequate DHA/EPA supplementation, joint health focused diets
Regular exercise	Suitable daily exercise, case specific exercises
Physical rehabilitation	Tailored rehabilitation program for muscle strength and joint support
Lifestyle adjustments	Changes for mobility support and injury prevention
Pain management	NSAIDs with individualized multimodal pain management plan
**Secondary treatment considerations**	
Pharmaceutical medications	Pregabalin/Gabapentin, Anti-NGFmAb
Nutraceutical supplements	Cannabinoids, chondroprotective joint health support (DMOAD)
Modalities	Tailored supportive modalities (see Table 6)
Interventional modalities	Joint injections, steroid epidural

### Coast Stage 4 (Severe OA)

Stage 4 refers to the advanced stage of OA in which patients demonstrate significant clinical signs and a higher level of dysfunction and pain. The signs are obvious, constantly present, and are significantly affecting the QoL of the dog. Those signs include severely abnormal limb loading and shifting of weight distribution with a reluctance and restlessness when standing; significant lameness with a reluctance to move and marked difficulties in rising and laying down. A limited ROM with crepitus, joint thickening, anatomical misalignment, and advanced muscle atrophy can be seen. Diagnostic imaging will show advanced osteophytes and signs of bone remodeling (10).

#### Treatment Goals

At Stage 4, the treatment goals are often very individual to effectively treat the multi-facetted pain states and often require a tailored level or expectation of mobility for the specific patient based on both the patient and client. The focus in this stage is the continuing assessment and adequate improvements/maintenance of QoL, including support for both owners and patient.

#### Prioritized Treatments

**Client education** includes all the topics mentioned in the general treatment recommendations for nutrition, joint health focused diet, omega 3 FA, weight optimization, exercise, and regular reassessments. Stage 3 specific education recommendations also apply. Specific Stage 4 focus points for education would be the impact on QoL as the disease progresses, as well as the importance of appropriate pain management and pain assessments. Regular orthopedic assessments should be emphasized to allow for tracking musculoskeletal changes and treatment results. Muscle wasting is a large concern especially for seniors that are already challenged with sarcopenia (29). Maintaining and possibly building muscle mass is one of the priorities for these patients. Creating a regular exercise and activity schedule that can be modified depending on the health of the dog is crucial. Regular, short, but frequent, low impact walks, and exercise (to tolerance of patient) even at this advanced stage are very important for preservation of mobility, physical and mental health.

At Stage 4, a **formal rehabilitation program** designed by a rehabilitation practitioner is highly recommended if logistics allow. Rehabilitation can provide assessments and discussions about QoL as well as appropriately assess and revise the pain management plan in collaboration with the family veterinarian. The owner often requires advanced lifestyle and home modifications to adjust to their pet's level of disability. Based on the same principles described in Stage 3, a rehabilitation team will create an individualized program for the patient that may include targeted therapeutic exercises which focus on core strength and posture, maintaining or gaining range of motion, improving overall physical fitness, and strengthening the musculature that is required to provide stability for osteoarthritic joints. This often includes a combination of a home exercise plan in addition to the formal in-clinic rehabilitation program and considers the lifestyle and ability of the owners.

**Lifestyle and household modifications** play an important role at this advanced stage and are similar to the modifications mentioned in Stage 3. These modifications focus on preventing any slipping and injuries and providing more comfort for the dog to ensure that QoL is maintained. Examples are the same as in Stage 3 with the addition of assistive mobility devices that may be helpful based upon the individual case situation. Supporting ongoing environmental enrichment and promoting the human-animal bond plays a role here.

For **pain management** the use of **NSAIDs** continues to be most highly recommended at this stage to keep the patient comfortable. If no co-existing diseases are present, lifelong administration is necessary. As patients are often older at this stage of disease, it is important to continue to monitor for the development of other diseases (kidney, liver, cancer) by regular bloodwork assessments and physical examinations. When NSAIDs are initiated, the same protocol as described in stage 2 and 3 applies. Other anti-inflammatory options may need to be discussed when dogs at this advanced stage have co-existing disease that prevent regular NSAID use. A discussion with owners may be initated to address QoL with aspect of efficacy of NSAIDs over risks of adverse events when no other treatment options provide adequate pain relief to prevent suffering of the animal.

**Anti-NGF monoclonal antibody (mAb)** is not yet available in Canada at the time of the preparation of this document, however we have included it in the guidelines due to its recent Canadian label approval (Feb 2021). Anti-NGF mAb has demonstrated potential in research and there has been clinical experience in the European market for use in late-stage OA (71–75). Nerve Growth Factor (NGF) and inflammatory mediators (cytokines, prostaglandins, etc.) play an important role as pain initiators and nerve sensitization in chronic pain (50, 76, 77). NGF is largely responsible for the neurogenic inflammation component in chronicity and severity of pain and, it regulates pain through nociceptor sensitization. The mechanisms of NGF on the pain signaling pathway are complex, involving various other receptors and are in parts responsible for the development of neuropathic pain and pain modulation peripherally and in dorsal root ganglion. Anti-NGF mAb blocks NGF from binding to the tropomyosin-related kinase receptor (TrkA) and p75 neurotropin receptor (NTR), subsequently inhibiting the pain signaling pathway potentially treating and slowing down peripheral nerve sensitization (78–80). It has shown to provide OA pain relief over the period of about 4 weeks after a single subcutaneous injection. The safety profile of bedinvetmab, the first anti-NGF mAb to be commercialized for dogs, appears to be high (75). Mild reactions at the injection site (e.g., swelling and heat) may uncommonly be observed. There are no safety data on the concurrent long-term use of NSAIDs and bedinvetmab in dogs. In clinical trials in humans, this has been reported as a potential source of rapidly progressive OA, the incidence increasing with high doses and in those human patients that received long-term (more than 90 days) NSAIDs concomitantly with an anti-NGF mAb (81). Dogs have no reported equivalent of the human rapidly progressive OA.

Once available, Anti-NGF mAb would be recommended as a core treatment for Stage 4 in particular (and possibly earlier) if the pain is refractory to treatment, suggesting that a neurogenic component from nerve hypersensitivity is present. The potential for Anti-NGF mAb to specifically treat the neuropathic or neurogenic component is promising and can be well-incorporated into a multimodal approach. A recent multicentre prospective efficacy study in clinical canine OA patients showed promising results as an additional option in the treatment of pain with seemingly remarkable safety profile. After 3 months of comparative study between a placebo (*n* = 146) and bedinvetmab (*n* = 141) with a treatment success rate (as defined by study criteria) varying from 50% (day 14) to 67.9% (day 56), the treatment success rate stabilized at about 75% over the continuation phase (up to day 252) (75). Clinical experience in the future will give more insights into this medication for OA in dogs as part of a multimodal treatment plan.

#### Secondary Treatment Options: (Stage 4)

Secondary treatment options are usually needed at Stage 3 and 4 due to the difficult characteristics of OA pain. The pain experience is unique for every individual, and accordingly as is their response to treatment(s). Factors including a patient's personality, receptor genetics, metabolism, degree and mechanisms of peripheral and central sensitization, which all serve to emphasize the importance of tailoring treatments to an individual patient.

However, the multimodal approach can be confusing due to a number of factors. Often the sheer number of treatment options can present challenges, the wrong application of therapies, a lack of understanding of the mechanismus of pain or modality, limited evidence, and the high variation in individual response in efficacy.

Most of the secondary treatment options could be considered in stage 3 or 4, as the order on what, when and how to introduce a new medication or modality to the multimodal approach is dependent on the individual dog, owner, veterinarian, and availability (of modality).

As mentioned previously, the secondary therapies did not receive unanimous support from the panel, the lack of support or difference in opinion often arose due to concerns in prioritization, variability or lack of scientific evidence, lack of experience with therapy and lack of clinical experience. Thus, each treatment below is presented in the context that limitations are present, and thus using clinical judgement to conduct a risk:benefit analysis for therapy is important prior to using it in a patient.

In this section a brief summary of options for Stage 4 specific is provided. The secondary treatment options recommended at Stage 3 apply and may have already been introduced.

- If gabapentin has been ineffective, a switch to **pregabalin** can be made based on clinical experience.
- After introducing pregabalin/gabapentin in Stage 3 in cases with presumed neurogenic/neuropathic hyperexcitability component of the pain, some panelists turn to amantadine as a third line treatment option. Evidence for efficacy in OA is limited, the only paper available provides questionable evidence of its effectiveness (82). Nevertheless, the mechanism of action of blocking the NMDA receptor may warrant its use in cases with pain hypersensitivity in conjunction with other pain medications. With similar evidence, **tramadol** can also be considered in association with an NSAID (61). See detailed description below.
- Some panelists would maintain photobiomodulation, acupuncture and PEMF therapy as supportive modality in the multimodal approach. See detailed description below.
- Some panelists would consider joint injections for joints that are refractory to treatment. See detailed description below.
- Some panelists would consider steroid epidural if indicated, especially for severe lumbosacral pain in conjunction with significant hind-end weakness. See detailed description below.
- Some panelists would consider starting or continuing cannabinoid medicine with appropriate veterinary oversight. See detailed description below.
- Some panelists would consider shockwave therapy (83, 84) as an added physical therapy modality. See detailed description below.
- Even though surgical intervention (arthroscopy, arthrodesis, *etc*.) was beyond the scope of this article, it is important to note that it may be warranted and considered in some cases of both Stage 3 and 4 to provide the needed relief of discomfort, following full recovery from said surgery. An informed discussion of the impact of surgery on both advantages and potential risks and disadvantages (including slower and less comfortable recovery period) is necessary, when performing surgery on an already heavily sensitized joint in a COAST OA stage 4 dog.

A summary of stage 4 treatment recommendations is provided in Table 5.

**Table 5:** Summarized core and secondary treatment recommendations for COAST Stage 4.

**STAGE 4**	
**Core treatment recommendations**	
Client education	QoL discussion and pain management, regular assessment, owner support
Weight optimization and nutrition	Adequate DHA/EPA supplementation, joint health focused diets
Regular exercise	Suitable daily exercise, case specific exercises
Physical rehabilitation	Tailored rehabilitation program for muscle strength and joint support, mental stimulation and QoL support
Lifestyle adjustments	Mobility and QoL support, injury prevention
Pain Management	NSAIDs, anti NGF mAb, individualized multimodal pain management plan
**Secondary treatment considerations**	
Pharmaceutical medications	Pregabalin/Gabapentin, Amantadine
Nutraceutical supplements	Cannabinoids, chondroprotective joint health support (DMOAD)
Modalities	Tailored supportive modalities (see Table 6)
Interventional modalities	Joint injections, steroid epidural

### Secondary Treatment Options for COAST Stages 3 and 4

The below information entails Stage 3 and 4 secondary treatment options with more detailed information including MOA, supporting literature and dosing information. They are grouped in categories and not ranked in preference of treatment.

#### Pharmaceutical Options

**Gabapentin** is often used as a second line treatment in chronic pain, including OA, in conjunction with NSAIDs, and is commonly added when a neuropathic pain component is suspected (85). The complete mechanism of action of gabapentinoids (gabapentin and pregabalin) has not been fully elicited, but its primary mechanism of action involves the presynaptic inhibition of voltage-gated calcium channels, which in turn blocks calcium influx, that would have led to the release of excitatory neurotransmitters. Due to the inhibition of the excitatory neurotransmitters, there is a decrease in pain signaling across the synapsis. Voltage gated calcium channels (VGCC) are upregulated in a chronic neuropathic state and gabapentin may influence the number of available and active calcium channels. Gabapentin has other, less understood mechanisms of action. These mechanisms are the antagonism of (but not direct binding to) the N-methyl-D-aspartate (NMDA) receptor, also known to be a calcium channel when activated. The antinociceptive effects of gabapentinoids have further been described to be associated with the noradrenergic and serotonergic activity *via* the descending pain pathway (86, 87). For dogs, there are no currently licensed veterinary products in Canada. Studies on efficacy of gabapentin for pain have been disappointing, as the evidence that gabapentin is efficacious to treat pain, in particular inflammatory pain, is low. Clinically it has been noted that gabapentin seems to show better effects when the patient has a neuropathic hyperexcitability component to their pain (significant central upregulation or nerve related pain). Dogs in late-stage OA commonly have a neurogenic inflammatory and central sensitization component and may exhibit back pain due to posture abnormalities and muscle atrophy related to the ongoing OA. The role of both gabapentin and pregabalin would theoretically reduce those components (88), but the evidence for the clinical efficacy has yet to be proven for OA. Recommended dosing is controversial and may depend on age and health status of the dog as well as co-administered medications. A pharmacokinetic (PK) study in greyhounds after a single dose administration concluded a dose of 10–20 mg/kg TID is required to reach plasma levels that compare to adequate levels for pain relief in humans (89), but no canine studies have been able to establish the plasma levels that provide analgesia or the PK results after long-term use. Clinically a 5–10 mg/kg TID dose prevents the unwanted side effects of significant ataxia, sedation, and urinary incontinence. Even though these side effects may be transient, they commonly affect an owner's compliance, and the panel members generally refrain from escalating doses beyond this, in particular in older dogs with pre-existing hind end weakness. Gabapentin is rarely used as a sole analgesic in veterinary medicine, and until there is better efficacy data, it should be used based on an individual assessment as part of a multimodal regimen.

**Pregabalin** is the gabapentinoid, that has preferable PK and pharmacodynamic (PD) profile over gabapentin (90). The oral bioavailability and duration of action is superior to gabapentin, and the binding to the delta subunit of the voltage-gated calcium channel is stronger, showing higher efficacy in humans (91). The recommended dose based on a canine PK study is 3–5 mg/kg BID (90). In conjunction with an NSAID, pregabalin (and presumably gabapentin) appears to be more effective in human OA studies addressing both the inflammatory and central neuropathic aspects of chronic OA (92). Beyond the recommended dose, pregabalin can have similar side effects as Gabapentin in older dogs.

**Amantadine** was considered as a third line treatment in refractory pain cases by the panel. Amantadine is an NMDA receptor antagonist and has the potential to be effective in reducing the wind-up effect in patients that show signs of central sensitization (refractory pain despite treatment, sensitive to touch). However, based on the current understanding of the mechanism of action, it does not likely work as a sole analgesic, and is usually recommended to be used in conjunction with an NSAID. Currently there is only one study assessing the efficacy of amantadine in OA pain in dogs (in addition to meloxicam, at a dosage of 3–5 mg/kg SID PO), and it showed incomplete and questionable beneficial treatment effects with a 3 week delay in onset (82). The current dose recommendations for dogs are 3–5 mg/kg BID which are based on a combination of a PK study that involved five Greyhounds (93) and extrapolations from human data. Due to the shorter T_1/2_life in dogs, the historically suggested once daily dose has been adjusted to twice daily (every 12 h) in dogs (93, 94). The PD effects, in particular the efficacy for pain, and adequate plasma levels that would be needed for analgesia have not been established and therefore the evidence for the use of amantadine as a pain medication in dogs with OA is low to non-existent. A study to determine the effectiveness of amantadine for pain in veterinary species is needed. Side effects are usually reduced appetite or vomiting, which in parts may be due to the bad taste of the formulation.

**Acetaminophen** has been infrequently suggested as a pain medication for dogs with OA. Under the name paracetamol it is more commonly used in Europe. Acetaminophen has a unique mechanism of action related to the endocannabinoid system (ECS). It produces a metabolite (N-arachidonoylphenolamine), which inhibits the enzymatic (FAAH) breakdown of anandamide, inhibits COX1 & 2, and is a TRPV1 agonist. This metabolism pathway may be species-specific and dose dependent but is a promising therapeutic avenue and reflects the interaction of the ECS in a variety of mechanisms of action of pain medications. Clinically human studies repeatedly suggest NSAIDs are superior for pain relief in OA (95) concluding acetaminophen to only play a role in the early-stage mild OA pain relief. Experimental research with induced synovitis study in dogs confirmed that the NSAID carprofen was superior to an acetaminophen-codeine product and that both the PK and PD of acetaminophen may not be sufficient for adequate pain relief and improvement of function (lameness) (96). It has to be noted that the study focused on the anti-inflammatory capacity of both treatments over a very short period of time (9 h) in a chemically-induced model. A long-term clinical study in dogs with OA would be needed to gain more insights, including its side effects and potential long-term effects on liver.

**Tramadol**. The role of tramadol in treatment of chronic pain has been controversial. Its mechanism of action in dogs is mainly *via* the descending pathway by means of norepinephrine and serotonin re-uptake inhibition. This descending pathway does play an important role in modulating the ascending pain signals. Tramadol seems to have a lack of measurable efficacy for OA in dogs as a sole agent (97, 98). In both studies, the duration of treatment was unusual short and the OA stage advanced (radiographically present) [10 days: Budsberg et al. (98); 14 days: Malek et al. (97)]. The analgesic efficacy of a NSAID-tramadol combination looked more advantageous over a 4-month period, including a 4-week daily initial regimen administration (61). The PK profile of tramadol is also not ideal in dogs (99), making a slow-release formulation (5–10 mg/kg PO daily) more attractive. Long-term use has been reported to have a decrease in effect (94). The concerns of gastric adverse effects in conjunction with NSAIDs due to the serotonin modulating gastric acid secretion and contributing to gastric lesions, have been assessed with no evidence to detect any deleterious effects (61, 100). Further studies are needed to investigate the effects of tramadol, as there may be emotional benefits contributing to pain control and QoL through the serotonin/dopamine and norepinephrine pathways (requiring more exposure to treatment to induce changes). These pathways on the other hand may also contribute to the negative behavioral side effects that one may see in some (senior) dogs. Based on current literature, tramadol plays a minimal role in the treatment of OA in dogs. More research is needed to further assess tramadol and its role in chronic pain in dogs.

#### Nutraceutical Options

With the recognition that pet owners are increasingly looking for botanical and “more natural” treatment options, as well as an increase in interest from the scientific veterinary community in the nutritional and medicinal use of herbal medicinal products, the expert group felt it important to include products and ingredients that have appropriate studies and evidence for OA treatment (101, 102). The list is not complete but includes some of the more common product groups.

It is important to recognize that nutraceutical combination products fall under the category of Animal Health Products, which are very differently regulated than the pharmaceutical industry (103), currently not requiring research or safety studies, but also cannot claim therapeutic benefits. When considering a specific product, it is important to assess specific ingredients and their concentrations. A certificate of analysis can be obtained, that shows a product is free of contaminants like residual solvents, heavy metals, microbials, pesticides, or fungus. Ideally a company can also be transparent about quality of product, source of ingredients and manufacturing standards. A natural product number (NPN), that is provided for a licensed natural health product assessed by Health Canada to be deemed safe, effective and of high quality, adds to assurance of quality. To date there is little information about the stability of products, ingredient interaction, bioavailability or PK profile, including dosing for most natural health products (103). Another area of needed research is the effects of using certain nutraceutical products together with other nutraceutical or pharmaceutical products, especially when the mechanism of actions works on similar pathways or receptors, or the metabolism is impacted. In general, a synergistic or additive effect is presumed, but scientifically not established for most products. Close monitoring for efficacy and adverse events is recommended as has been previously suggested for multimodal pain management.

**b.1 Cannabinoids**. The endocannabinoidome system is involved in almost all aspects of the ascending and descending pain pathway at all major signaling points including the periphery, spinal cord and CNS. The endocannabinoidome system extends from the ECS system (receptors (CB1 and 2), enzymes and ligands) to other classic receptor systems that are part of the pain pathway (opioid, TRPV, serotonin, prostaglandins, etc.). Cannabinoids have been shown to play a role in neurogenic and inflammatory pain by a variety of mechanism of actions on various receptors and pathways (104). The use of cannabinoids in veterinary medicine is still relatively new, nevertheless there have been multiple clinical trials published that show promising results for its efficacy for pain relief of OA (70, 105–110). The existing studies conducted have been product specific, in that the researched product has a specific cannabinoid and terpenoid profile. Unfortunately, this makes it challenging to extrapolate and interpret the results of PK and PD toward other comparable products (69, 70, 107, 111). The safety profile needs further investigation, particularly with regards to causes of liver enzyme elevation and its effects on liver function (107, 110). Current regulations in veterinary medicine make it difficult for veterinarians to support owners with finding a consistent product that can be safely used in their pet. From clinical experience, a cannabidiol (CBD) isolate product may not provide adequate pain relief in moderate to severe OA cases but can be useful in mild cases (110, 112). A full spectrum CBD|THC product has been shown to be more effective in advanced pain cases based on the nature of pain (inflammatory, immune-mediated, neurogenic). The role of THC/THCA and CBD/CBDA as a CB2 receptor agonist in more severe or immune mediated pain is still to be further investigated in dogs but would presume to play a role based on research from other species (113–115). Like other medications, cannabinoids used for OA pain should be accompanied by regular wellness evaluations, blood work and monitoring for patient response or adverse events, as synergistic effects can be noted when used together with other medications due to overlapping mechanism of actions and changes in metabolism (112).

**b.2.Chondroprotective agents**:

Due to the destruction of cartilage as part of the disease process in OA, the search for biological substances with the ability to restore the damaged connective tissues and protect the cartilage and chondrocytes is ongoing. These substances are considered chondroprotective agents and if effective are termed disease-modifying osteoarthritis drugs (DMOADs) (116). Currently there is still a discrepancy between *in-vitro* and *in-vivo* studies, in parts due to the lack of medications that have proven adequate oral bioavailability and distribution to site of cartilage. Commonly considered chondroprotective agents that have been assessed in clinical trials and research studies, although with often limited conclusive results, include glucosamine hydrochloride (or sulfate), chondroitin sulfate, avocado soybean unsaponifiables (ASUs), egg-shell membrane extract, sodium pentosan polysulfate (PPS), green lipped mussel extract, type II Collagen (UC-II), and elk antler velvet extract among others.

**b.2.1 Glucosamine and chondroitin** have been suggested to have chondroprotective effects and are commonly used for OA patients in both human and veterinary medicine. Yet, inconsistent study design among studies has resulted in limited and conflicting results (117), causing questioning of their actual efficacy in veterinary species. In brief, based on *in-vitro* data, glucosamine is partly responsible for the regulation of collagen synthesis in cartilage and it contributes to glycosaminoglycan and proteoglycan synthesis (118, 119). Chondroitin sulfate is a sulfated glycosaminoglycan and contributes to extracellular matrix of cartilage and adds resistance and elasticity to the cartilage (120). The role of chondroitin sulfate is the inhibition of specific destructive enzymes in joint fluid and cartilage and, like glucosamine, contributes to the synthesis of glycosaminoglycans and proteoglycans (119, 120). The biggest challenge that both chondroitin sulfate and glucosamine face are low oral bioavailability and inconsistencies in product formulation (strength, form for glucosamine as sulfate vs. hydrochloride, and other ingredients added to the product), both contribute to the inconsistent results found in efficacy evaluation in literature and clinics. Most veterinary products contain glucosamine hydrochloride, which has significantly less bioavailability in humans than glucosamine sulfate. Pharmacokinetic studies are limited (121, 122) and the dosing recommendations of 15–30 mg/kg seem arbitrary and have not been established based on pharmacological evidence. The study performed by Adebowale et al. (123) demonstrated an oral bioavailability of 12% for glucosamine hydrochloride and 5% for chondroitin sulfate. Despite the differences in clinical studies from a point of view of design, products and results, there have been some well-conducted studies that provide more insight into the use of glucosamine and chondroitin (124–131). These studies evaluated joint function, comfort of the patient, and the overall safety profile of the supplement. The various outcome measures were aimed at establishing the potential anti-inflammatory and presumed mechanical improvements due to advanced cartilage function, with some positive results. The follow-up ranged from 2 to 6 months, with dosing around 40–62.5 mg/kg/d for glucosamine hydrochloride and 12–50 mg/kg/d for chondroitin sulfate in dogs, doubled for cats (132). No improvement was observed with objective outcomes in any study, and mild improvement was observed with non-validated subjective clinical scoring at three time-points in one study [Day 90, 120, and 150 (129)] and in one time-point in another study [Day 70 (127)]. Little is known about the actual effects of glucosamine and chondroitin at the level of the joint in either late or early stages of OA (133, 134). Canapp et al. showed evidence of protective effects of glucosamine hydrochloride with chondroitin sulfate at the level of joint, when given preemptively in an induced synovitis study (135), which would be different from OA. Despite the concerns about the lack of evidence in this category, owners and veterinarians alike continue to use or recommend products that contain glucosamine and chondroitin, often due to the high safety profile of most products. Education is important to clarify understanding and expectations of these products, in addition further studies including systematic review and metanalysis are required, to help resolve concerns of bioavailability and ultimately, efficacy.

**b.2.2 Avocado soybean unsaponifiable (ASUs)** is a mixture of the unsaponifiable fractions of one-third avocado oil and two-third soybean oil, that had promising effects for OA as a nutraceutical (136). The mechanism of action has been suggested to be inhibitory on interleukin-1 (IL-1) and stimulating on collagen synthesis based on *in-vitro* articular chondrocyte culture study (137). A potent inhibition of IL-8 and PGE_2_ has also been suggested (138). Cartilage repair may be promoted by its action on subchondral bone osteoblasts by preventing the osteoarthritic osteoblast-induced inhibition of matrix molecule production. Clinical studies in human OA with a focus on pain reduction outcomes show positive but limited evidence (139). A structural assessment study was done in a canine cruciate model, which demonstrated that ASUs reduce the development of early osteoarthritic cartilage and subchondral bone lesions. The suggested mode of action was mediated by the inhibition of inducible nitric oxide synthase and matrix metalloproteinase 13 (MMP-13), both key mediators of structural changes in canine OA (140). Dosing used in this study was 10 mkg/kg/day over 8 weeks. A study by Altinel et al. (141) evaluated ASU administration based on joint fluids and saw an increased levels of transforming growth factor beta 1 and 2 (TGF-ß1 and 2), both considered to be associated with the chondrocyte production of collagen and proteoglycans. Dosing for this study was 300 mg/dog SID, which translated to about 12 mg/kg. One clinical trial was conducted to assesses the efficacy of ASU in conjunction with glucosamine and chondroitin (131), not showing a significant difference in results, which may possibly be due to relatively low dosing (2.5–4.5 mg/kg/d). Overall, the evidence for ASU having beneficial effects in canine OA is limited but so far positive for both symptom relief and potential chondroprotective effects, although product differences need to be considered.

**b.2.3. Egg-shell membrane** (ESM) is the mesh-like bilayered substance that is found between the calcified shell and the albumin in chicken eggs. It is primarily composed of fibrous proteins such as collagen type I, keratin and elastin and glycosaminoglycans (142, 143). Egg-shell membrane extract has been evaluated *in-vitro* and showed an inhibition of IL1 β and tumor necrosis factor alpha (TNFα) (142, 144). A clinical study with a commercial product showed some positive effects on symptom relief that was detectable after 1 week and lasted throughout the study period of 6 weeks but lacked the statistical significance. The study further detected a change in serum levels of the cartilage degradation biomarker, c-terminal cross-linked telopeptide of type-II collagen (CTX-II) and concluded to a chondroprotective aspect (145). Dosing in the study was 300 mg/dog daily (equivalent to about 13.5 mg/kg daily), extrapolated from the effective studied human dose of 500 mg/day). Another study that examined the effect of a commercial ESM product in dogs with hip dysplasia found a clinical benefit of symptom relief at 15 mg/kg/day (143). Finally, another commercial ESM product was recently tested (146), and the dosing regimen was as per package (soft chews) recommendations, but a mg/kg dosing information was not made available. If the changes were in favor of the treated group, the differences did not reach statistical significance. Egg shell membrane supplements may be an option for symptom relief, however its role in chondroprotective measures and its pharmacokinetic profile require future studies to be completed.

**b.2.4. Systemic DMOADs – Pentosan polysulfate sodium** (PPS) is a polysulfate ester of xylan, prepared semi synthetically from beechwood plant material and is structurally similar to glycosaminoglycan (147). The mechanism of action may be a stimulation of hyaluronic acid and glycosaminoglycan synthesis, inhibition of proteolytic enzymes including metalloproteinases, and free radical scavenging as well as reduction of cytokine activity and osteoclast differentiation (147–149). The sodium derivative of PPS [sodium pentosan polysulphate (NaPPS)] has been available in veterinary medicine, is administered in the form of a subcutaneous injection and has been approved as a DMOAD, but its efficacy has not been fully established in the literature and remains controversial among clinicians. A canine post cruciate surgery study saw a faster recovery in one outcome measure compared to the placebo (150) and a clinical human study with knee OA found significant improvements in symptom relief compared to the placebo group (147). Yet clinically the results in dogs are inconsistent. Some patients demonstrate mild to moderate improvement, while others show no response. More studies are needed to assess the clinical and chondroprotective effects in dogs.

**b.3**. ***Boswellia Serrata*** (also known as “true” frankincense) has been included in many anti-arthritic joint supplements and has been shown to have anti-inflammatory properties in published studies (151). It has been traditionally used for centuries for this purpose (152). The active ingredient from the tree is the oleo-gum resin, and it is harvested by collecting the sap of the tree, then it is processed for use (stored, solidified and graded) (151). *Boswellia* resin is a traditional remedy for multiple ailments, but its anti-inflammatory properties held therapeutic interest and have been further explored. One of the mechanisms of action for its anti-inflammatory property is the inhibition of leukotrine (5HETE and leukotrine B4) synthesis by blocking the 5-lipoxygenase. It also has been shown to reduce glycosaminoglycan degradation, inhibition of TNF α and IL-1 β *in-vitro* (153). As with many of the other nutraceutical ingredients, the oral bioavailability in dogs can be challenging and species-specific PK studies are needed (154). Product formulation and manufacturing techniques also play a role in efficacy and safety. There have been some clinical studies in dogs (155) and humans (156), and *Boswellia* seems to have a wide safety range, based on acute and chronic toxicity and safety studies. However, dose determination research is needed, as the dose in one canine study was 40 mg/kg, but others have suggested 50–100 mg/kg once daily (155).

**Other and combination products:** Products that combine different nutraceuticals are available and popular among pet owners. There are commercially available products as well as veterinary specific products that have been scientifically assessed with promising results: omega-3 FA, including green-lipped mussel, products are the most recognized (34, 35). *Curcuma* efficacy alone (157), or in combination with collagen and green tea extract in an enriched therapeutic diet (158) did not show clear results. However, multi-herbal, omega-3, glucosamine combination in two different formulations (124, 159) was more convincing. Specific ingredients in combination products with promising efficacy shown in studies include epiitalis (160, 161), undenatured type II collagen (UC-II) either alone (128, 162–164) or in combination with other chondroprotective ingredients (128, 129, 163), and warrant mentioning and further research.

Finally, promising natural health products, such as elk-velvet antler (165) or the *Brachystemma calicinum* D. don Chinese plant (166, 167), that have attractive analgesic benefits currently have a minor role in commercialization due to their controversy in safety and quality control production.

#### Other Modalities

Under this category, there are specific modalities that are commonly used clinically in OA patients and have been scientifically evaluated (Table 6). We provided a summary but encourage the reader to further their own knowledge with additional research on available studies, bias, risks, side effects, techniques, required level of training or certification process, and benefits. These details were beyond the scope of this paper, but are important information, when considering the different modalities. The list of OA related modalities presented in these guidelines is not exhaustive, additional modalities may be considered by experienced and trained practitioners (including osteopathy, chiropractice, canine massages, cryotherapy, therapeutic ultrasound among others), and should also be based on available evidence that results from appropriate study design.

**Table 6:** Potential modalities discussed in this paper that may be added to an OA treatment plan to support the individual patient.

**Potential modalities to support OA treatment plan**
Acupuncture
Photobiomodulation
Pulsed ElectroMagnetic Field therapy (PEMF)
Extracorporeal Shock Wave therapy (ESWT)
Joint injections
Steroid epidural

**Photobiomodulation (Laser) treatments** can potentially be beneficial for some patients, using appropriate settings for specific tissues or conditions. There have been significant knowledge gains in the field of laser therapy over the last 10 years and it is important to understand the technicalities to assure that the targeted tissue depth is reached, as it will vary depending on tissue (168, 169). The mechanism of action of Laser (light amplification by stimulated emission of radiation) is on a cellular level *via* photobiomodulation. Investigators have shown that laser application on tissue has multiple effects including an increase in angiogenesis, neurite extension, normalization of ion channels, stabilization of the cellular membrane, and other cellular changes (170), but the most recognized mechanism is the nitric oxide (NO) interaction in the cytochrome C system leading to improved ATP utilization and production. Reducing inflammation and edema through means of IL-1 reduction, acceleration of leucocyte, and inhibition of PG synthesis have also been discussed. In veterinary medicine Low Level Laser therapy (LLLT) can be useful in the treatment of musculoskeletal pain (170) using either a class IIIB or IV laser. Usually, wavelengths in the therapeutic window between 600 and 1,100 nm are used, with adequate penetration into tissue requiring a minimum of 800 nm. Laser therapy can reduce muscle tension when used for surface area application, however treatment of OA *via* intra-articular penetration of the laser beam requires a higher power (higher than 4–8 J cm^2^) or a longer duration of treatment. The risk of burns at the higher wavelengths are avoided by constant movement of the probe, extra precautions with darker skin/fur animals as the absorbed light in these patients may produce warm energy. At times shaving thicker fur may improve wavelength penetration. Laser therapy is used as an integral part of rehabilitation protocols (64, 170, 171) and is commonly used as an add on modality to an overall sound treatment plan. It appears that with better understanding of laser therapy and better designed studies, the knowledge for its usefulness in the treatment of OA has improved (64, 169, 171–174) but it is not yet conclusive (175). It will require further extensive and appropriate investigations to answer its benefits, risks, limitations and settle the strong controversy surrounding this modality.

**Acupuncture** can be an effective conservative treatment for neuro- and musculoskeletal pain conditions including OA and is recommended as an adjunct therapy within the multimodal approach (176). Various studies have been published in dogs with OA with different outcome measures and assessments, as well as acupuncture techniques and results. The changes that have been noted seem subtle but positive, although not always statistically significant. Acupuncture is a modality that seems to have an individual response irrelevant of whether a Western or Eastern approach is used. Extensive human studies have shown a beneficial effect of acupuncture in the treatment of OA (177), but metanalyses are not always conclusive and the claimed small analgesic effect cannot be clearly distinguished from bias (178). Veterinary studies are not different (65), in parts due to the difficulties in pain assessment and the challenges of standardizing a treatment protocol. The differentiation of electroacupuncture vs. dry needle acupuncture is one of the questions that would be interesting to have answered. Acupuncture is a highly individualized therapy that is commonly used within a rehabilitation program or as part of palliative care, continues to gain popularity within the veterinary community.

**Mechanical stimulation: Pulsed ElectroMagnetic Field therapy** (PEMF) **and Extracorporeal Shock Wave Therapy** (ESWT). **PEMF** is an emerging area of interest for OA treatment in both human and veterinary medicine (179). PEMF utilizes frequencies at the low end of the electromagnetic spectrum (6–500 zH), which stimulate biological effects on a cellular level. The mechanism of action remains not fully understood but potential mechanism of actions of PEMF are the stimulation of chondrocyte proliferation and differentiation, as well as extracellular matrix synthesis. PEMF can cause a decrease in inflammatory cell infiltration, reduction in immuno-positive cells to IL-1β, decrease in TNF-α, and increase TGF-β 1 (promoting cartilage repair). PEMF shows promising results in both *in-vitro* and *in-vivo* studies to provide pain relief, improved function and slowing down the progression of OA (180, 181). Although literature is readily available, the quality of veterinary studies on PEMF are still limited (182) and more research needs to be conducted. As an adjunct, non-invasive therapy, this modality will likely play an increasing role in clinics and, especially for in-home use in form of commercially available loops, discs, and mats (182). **ESWT** is a special, non-linear type of pressure wave with a short rise time (around 10 μs) and a frequency ranging from 16 to 20 MHz. Different ESWT units are available with different wave forms (radial, piezioelectric and electrohydraulic) which will lead to different tissue penetration of the acoustic wave. Extensive knowledge and training is needed for appropriate application of this modality. In particular the electrohydraulic waves will require sedation of the patient, potentially part of the reasons why this modality is less mainstream in veterinary practice. Several studies have demonstrating attractive value of ESWT in managing canine musculoskeletal alterations, mostly OA, either for stifle (183), shoulder (184) or hip (83, 185) joints.

#### Joint Injections

As part of “regenerative medicine,” joint injections of “orthobiologics” or drugs have been explored for local pain relief of a specific joint. Severe OA in the elbow, shoulder or hip can be difficult to treat to provide adequate comfort. Injections of hyaluronic acid (HA), mesenchymal stem cells (MSC) or platelet-rich plasma (PRP), autologous protein solution (APS) have been proposed and investigated. This panel has agreed that as of now, most of the research in canines has been published with PRP injections (see below) and is therefore currently the recommended choice, if a joint injection is considered. Stem cell injections have some chondroprotective and regenerative potential, however, are still considered to be in their infancy. Injections of HA and steroids are usually reserved for palliative cases as the pain relief may show benefits for symptom relief, but the chondro-destructive potential remains controversial for steroid injections (186). The use of HA/steroid joint injections in human OA appears to be favorable in the literature due to the improvement of symptoms as well as the joint lubricating effects of HA but has a relative short duration and a limited number of injections per year. It remains quite uncommon in canine medicine (187, 188). The use of HA has been studied either in experimental models (189) or in clinical use in OA dogs (190) with positive outcomes over an appropriate (several months) length of time (67, 68, 191). It has been previously suggested that intraarticular HA would be more effective in dogs with mild to moderate OA than in those with severe OA (189). The combination of intraarticular HA and triamcinolone looks efficient too (187).

**Platelet-rich plasma (PRP)** is an orthobiologic composed mainly of platelets, which in turn will release growth factors to then stimulate other cytokines and chemokines. Derivatives include autologous platelets concentrate (APC) and APS. The growth factors (platelet-derived growth factor (PDGF), transforming growth factor β 1 &2 (TGF-β 1 &2), vascular endothelial growth factor (VEGF), basic fibroblastic growth factor (bFGF) and epidermal grow factor (EGF) among others) are the driving forces as important bioactive compounds contributing to wound healing by enhancing cellular migration, cellular proliferation, angiogenesis, and matrix deposition, which in turn may counteract cartilage destruction (192, 193). Platelet preparation systems vary in their ability to concentrate platelets, as well as select beneficial cells over unwanted cells like RBC, leukocytes, neutrophiles (193, 194). The ideal concentrations of platelets and WBCs are unknown, and likely depend on the type and chronicity of the injury. In the literature, the high variability in PRP preparations may be the reason for inconsistent results, but emerging clinical and *in vitro* studies, and clinical experience seem promising. PRP preparation can be done by specific systems (commercial gravity systems or centrifuge systems), that can be acquired. The protocol involves a blood draw from the patient which in turn is spun down in a centrifuge to separate RBC and plasma from the platelet/WBC layer. In some instances, a second centrifugation is recommended for further platelet concentration. The final product is then injected meticulously aseptically into the joint to avoid any infection. Duration of pain relief may last from 3 to 12 months and has been reported in OA dogs for PRP alone (195–199) or associated to HA (67) or physical therapy, showing longer duration of analgesia (197), APC (200, 201), APS (187, 202, 203). Most interestingly, PRP may have the potential to slow the progression of the disease, as suggested by a recent metanalysis including 1,251 animals (19 studies on rodents, 13 on rabbits, 4 on horses, one on goats, and 7 on dogs) (204). The disease-modifying effects (DMOAD) were present in 68% of the studies (beneficial clinical effects in 80%) and included attenuating cartilage damage progression, and reducing synovial inflammation, coupled with changes in biomarker levels.

The comparison between intraarticular HA, APC, triamcinolone, and stanozolol supports more prolonged analgesic benefits, with lower variation in results for HA and APC (205). Several reports of beneficial analgesia exist after intraarticular MSC injection (206–212). However, these reports present methodological drawbacks, such as absence of standardization in MSC preparation, either limited power of analysis, and/or subjective outcome measures, or most often lack of a placebo control, to really state about the interest of intraarticular MSC injection. Finally, some anecdotal publications mention the use of intraarticular botulinum toxin A for pain management in OA dogs (213, 214), of Tin-117m (^117m^Sn-colloid) isotope radiopharmaceutical in canine elbow OA (215), and of intraarticular resiniferatoxin for long-term analgesia (216).

**Steroid epidural**: Senior dogs especially, but also young working dogs (i.e., German Shephard military or police dogs) may exhibit severe lumbosacral pain. This can be a primary disease, as some dog breeds are predisposed to it, but it also can be part of posture changes related to late-stage OA in hips or knees. The resulting progressive hind-end weakness due to avoidance of muscle usage caused by pain leads to significant mobility issues. An epidural injection of long-acting steroid can provide relief of a duration from 4 to 12 months (217, 218). For an epidural injection, the patient is heavily sedated, or in case of health-related concerns briefly anesthetized. The lumbosacral area is aseptically prepared and 0.1 mg/kg methylprednisolone acetate injected sterile. Complications appear to be rare in veterinary medicine and may relate primarily to sterility and trauma but have also been linked to the formulation (including preservatives and particulation). In human chronic spinal pain management, complications can be severe, and concerns have been raised specifically regarding the use of particulate steroids -like methylprednisoline- when applied epidurally (219).

## Summary

The Canadian OA treatment guidelines were created from a diverse group of experts, driven by the shared understanding of the need for providing direction for veterinarians on selecting appropriate therapies based on COAST stage for a patient experiencing OA. Due to the inflammatory nature, chronicity, potential neurogenic component and continued progression of the disease, OA requires a multimodal approach. The treatment options for OA are constantly evolving as new therapies and research emerge, and this document captures the current or soon to be arriving options for Canadian veterinarians in 2022. It aims to provide insights into the treatment choices of experts and is grouped based on consensus into core and secondary treatments.

The panel felt that for every OA patient, their multimodal plan should involve client education, a weight management plan, optimized nutrition including omega 3 fatty acids, exercise, and beginning in stage 2, pain management. Additional secondary therapies or modalities can then be layered on, based on OA stage, individual patient need and veterinarian or pet owner preference. A cautious and rigorous characterization of the pain syndrome affecting the patient must guide the veterinarian to the best choice of therapeutics. With the fundamental understanding that the multimodal approach should always be aimed at slowing the progression of OA, maintaining patient mobility and above all, maximizing patient comfort and QoL.

## Author Contributions

CM wrote the first draft of the manuscript. All authors contributed to the discussion for the content of this manuscript, wrote sections of the manuscript and contributed to the correctness of the content (in order of their authorship, besides ET, who added significant amount and is considered senior author on this manuscript), and manuscript revision, read, and approved the submitted version.

## Funding

Elanco Canada Limited supported the development of these OA guidelines as part of their commitment to education and the orthopedic health and pain management of dogs.

## Author Disclaimer

These guidelines and recommendations should not be construed as dictating an exclusive protocol, course of treatment, or procedure. They are considered to be a helpful informational summary of available treatment options with a consensus from experts with a critical evaluation of the literature and clinical expertise. Although every effort has been made to ensure the completeness and accuracy of the information provided herein, neither the authors nor Elanco Canada Limited assume any responsibility for the completeness or accuracy of the information. All information is provided “as is” without any warranties, either expressed or implied. As each case is different, veterinarians must base their decisions on the best available scientific evidence in conjunction with their own knowledge and experience.

## Conflict of Interest

CM is currently a Veterinary Consultant for Orthopedic Health and Pain for Elanco Canada Limited. The remaining authors declare that the research was conducted in the absence of any commercial or financial relationships that could be construed as a potential conflict of interest.

## Publisher's Note

All claims expressed in this article are solely those of the authors and do not necessarily represent those of their affiliated organizations, or those of the publisher, the editors and the reviewers. Any product that may be evaluated in this article, or claim that may be made by its manufacturer, is not guaranteed or endorsed by the publisher.

## Abbreviations

ALA: alpha lipoic acid
APC: autologous platelets concentrate
APS: autologous protein solution
ASU: avocado soybean unsaponifiables
bFGF: basic fibroplastic growth factor
CB1+2: cannabinoid receptor 1 and 2
CBD: cannabidiol
CBDa: acid form of cannabidiol
COAST: canine osteoarthritis staging tool
COX: cyclooxygenase enzyme
CTX-II: c-terminal cross-linked telopeptide of type-II collagen
DHA: docosahexaenoic acid
DMOAD: disease-modifying osteoarthritis drug
DPA: docosapentaenoic acid
ECS: endocannabinoid system
EGF: epidermal grow factor
EPA: eicosapentaenoic acid
EP4: E-type prostanoid receptor 4
ESM: eggshell membrane
ESWT: Extracorporeal Shock Wave Therapy
FA: fatty acids
FAAH: Fatty acid amide hydrolase
HA: hyaluronic acid
IL-1 or 8: interleukin-1 or 8
Laser: **l**ight **a**mplification by **s**timulated **e**mission of **r**adiation
LLLT: Low Level Laser therapy
LOAD: Liverpool osteoarthritis in dogs questionnaire
mAb: monoclonal antibody
MMP-13: matrix metalloproteinase 13
MSC: mesenchymal stem cells
NGF: nerve growth factor
NMDA: N-methyl-D-aspartate receptor
NO: nitric oxide
NPN: natural product number
NSAID: non-steroidal anti-inflammatory drug
NTR: neurotropin receptor
OA: osteoarthritis
PD: pharmacodynamics
PDGF: platelet-derived growth factor
PEMF: pulsed-electromagnetic field therapy
PG: prostaglandin
PK: pharmacokinetic
PPA: pentosan polysulfate sodium
PRP: platelet rich plasma
QoL: quality of life
ROM: range of motion
T1/2: half life
TGF-ß1 and 2: transforming growth factor beta
THC: tetrahydrocannabinol
THCa: acid form of tetrahydrocannabinol
TNFα: tumor necrosis factor alpha
TrkA: tropomyosin-related kinase receptor
TRPV: transient receptor potential cation channel—vanilloid
UC-II: type II Collagen
VEGF: vascular endothelial growth factor
VGCC: voltage gated calcium channel.
